# Population Distribution of Phosphate-solubilizing Microorganisms in Agricultural Soil

**DOI:** 10.1264/jsme2.ME21041

**Published:** 2022-03-26

**Authors:** Irnanda Aiko Fifi Djuuna, Saraswati Prabawardani, Maria Massora

**Affiliations:** 1 Department of Soil and Land Resources, Faculty of Agriculture The University of Papua, Gunung Salju St. Kampus UNIPA Amban Manokwari 98314 Papua Barat-Indonesia; 2 Department of Agronomy Faculty of Agriculture The University of Papua, Gunung Salju St. Kampus UNIPA Amban Manokwari 98314 Papua Barat-Indonesia; 3 Department of Biology, Faculty of Mathematics and Sciences The University of Papua, Gunung Salju St. Kampus UNIPA Amban Manokwari 98314 Papua Barat-Indonesia

**Keywords:** biofertilizers, soil microorganisms, phosphorus, Manokwari-Papua Barat

## Abstract

Phosphorus (P) is an essential macronutrient for plant growth and is mainly present in agricultural soil in unavailable forms. Phosphate-solubilizing microorganisms (PSMs) increase soil P availability. The objective of the present study was to assess the population and type of PSMs and their relationships with soil characteristics in the agricultural soil of Manokwari. Twenty-one composite soil samples (0–20‍ ‍cm) were collected at the rhizospheres of plants in the Prafi and Masni Districts. A dilution technique and plate count method on Pikovskayas agar medium were used to examine the PSM population, phosphate-solubilizing index (PSI), and various soil properties. The results obtained showed that the total population of phosphate-solubilizing bacteria ranged between 25×10^3^ and 550×10^3^ CFU g^–1^ of soil, while that of phosphate-solubilizing fungi was between 2.0×10^3^ and 5.0×10^3^ CFU g^–1^ of soil at all locations. The PSI of the isolates ranged between 1.1 to 3.6‍ ‍mm, with the most efficient and highest PSI being obtained for *Bacillus* sp. (strain 8) and the lowest for *Pseudomonas* sp. (strain 15). Six isolates found at all locations were identified at the genus level: *Chromobacterium* sp., *Pseudomonas* sp., *Bacillus* sp., *Micrococcus* sp., *Caulobacter* sp., and *Aspergillus* sp. A correlation was observed between the number of PSMs and the level of soil P availability and moisture content, indicating an increase in soil P availability with a greater abundance of PSMs in soil.

Soil microorganisms play an important role in the decomposition of organic matter and the transformation of soil nutrients used for plant growth and development. They are also crucially involved in soil P dynamics and P availability for plants (Richardson, 2001) as well as in pollutant bioremediation and the maintenance of soil productivity (Dong *et al.*, 2014; Luo *et al.*, 2015). Many agricultural soils represent a phosphate sink in which this element is not readily available to plants, but may still be recovered. Groups of phosphate-solubilizing microorganisms (PSMs) are currently the most common among soil microorganisms because they are used as biofertilizers and as one of the alternatives to increase the efficiency of phosphate fertilizers in order to overcome low phosphate availability in soil (Alori *et al.*, 2017; Kalayu, 2019; Nosheen *et al.*, 2021; Tian *et al.*, 2021). PSMs may dissolve unavailable phosphate, thereby increasing soil P availability. Since it is easily absorbed by plants, P may enhance crop yields if its previous level was a limiting factor. Furthermore, PSMs include different groups of microorganisms, which not only assimilate phosphorus from insoluble forms of phosphates, but also release a large portion of soluble phosphates at quantities that are in excess of requirements. PSMs have been shown to increase the bioavailability of soil insoluble phosphorus for plant use (Zhu *et al.*, 2011). This group of bacteria, fungi, and actinomycetes is normally found in agricultural soil, with larger populations of phosphate-solubilizing bacteria (PSB) being found in agricultural and rangeland soils (Yahya and Azawi, 1998). Among the phosphate-solubilizing fungi (PSF) commonly found in agricultural soil, such as *Penicillium* sp., *Mucor* sp. and *Aspergillus* sp. increased plant growth by 5–20% after inoculation (Gunes *et al.*, 2009). PSB have been shown to enhance the solubilization of insoluble P compounds through the release of low-molecular-weight organic acids and phosphatases (Rossolini *et al.*, 1998; Sahu and Jana, 2000). Zhang *et al.* (2020) reported that P availability increased with the amount of PSB in solubilizing organic P (or PSBop), while the number of PSF increased with the content of soil organic carbo (SOC) and produced organic acids by dissolving insoluble phosphate with a decrease in pH, the chelation of cations, and competition with phosphate on sorption sites in soil (Mardad *et al.*, 2013; Anand *et al.*, 2016). Many PSB are effective biofertilizers or biocontrol agents and are regarded as broad spectrum biofertilizers (Gupta, 2004). Due to the negative environmental impact of chemical fertilizers and the increasing cost of energy, the utilization of PSB is advantageous for sustainable agricultural practices (Gyaneshwar *et al.*, 2002). The use of PSMs as biofertilizers may also overcome the excess of phosphate in acidic soil; therefore, they are expected to become an alternative that reduces dependence on the excessive use of phosphate fertilizers. The population of PSMs in soil ranges between 10^4^ and 10^6^ g^–1^ of soil and they are mostly located in the rhizosphere. Among these microorganisms, bacteria are more effective at phosphorus solubilization than fungi (Alam *et al.*, 2002). Previous studies reported that among the whole microbial population in soil, PSB were responsible for between 1 and 50% of the P solubilization potential, whereas PSF only accounted for between 0.1 and 0.5% (Chen *et al.*, 2006). The population density of PSB ranged between 8×10^5^ and 5.33×10^9^ in the different rhizospheres of vegetable fields (Alia *et al.*, 2013), between 5.0×10^3^ and 7.5×10^6^ CFU g^–1^ of soil in Wamena soil (Suliasih and Widawati, 2005), and between 0 and 10^7^ cells g^–1^ soil in the soil of North Iran, with PSB accounting for 3.98% of the total population of bacteria (Fallah, A. *et al.*, 2006. Abundance and distribution of phosphate solubilizing bacteria and fungi in some soil samples from north of Iran. 18th World Congress of Soil Science. Philadelphia, Pennsylvania, USA July 9–15). However, their number is not sufficiently high to compete with other microbial species in the rhizosphere (Jain *et al.*, 2012). The population or proportions of these microbes vary between ecosystems due to the influence of complex biological factors. Many soil factors, such as the soil nutrient status, soil pH, moisture content, organic matter, and soil enzyme activities, also contribute to these variations. Therefore, the objectives of the present study were to (1) isolate and assess the number and distribution of PSMs in agricultural soil samples, particularly the rhizosphere of agricultural plants; (2) characterize the isolated microorganisms; and (3) assess their relationships with soil characteristics.

## Materials and Methods

### Study area

The Prafi and Masni Districts are located in the Southern Manokwari Region, Papua Barat in the Province of Indonesia. These areas have been developed as a Central Agricultural Area based on the Transmigration Program since 1979 and are classified as plain areas called Arfak Flakte (the Arfak Alluvial Plain). These two areas are fertile, and the major crops are paddy rice, legumes, horticultures, corn, and oil palm. The land use history of this area is mostly food crops (paddy rice and tuber crops) and vegetables as well as some plantation crops. Soil developed from alluvial soil that was dominated by coarse fractions on the surface and fine fractions in the subsoil. There are four soil types in these areas: Entisols, Histosols, Inceptisols, and Ultisols (Darmawanto, 1994).

### Collection of soil samples

Soil samples were collected at a depth of 0–20‍ ‍cm in the rhizosphere at sample points in several locations in the Prafi and Masni Districts of the Manokwari Region in October 2018. A composite soil sample was obtained by mixing 10 sub-samples collected at the rhizosphere of each plant with soil auger (diameter of 2.5 inches). Samples were placed into sterile containers and transported to the laboratory, where they were air-dried and crushed. A portion of each sample was sieved with a 2-mm sieve mesh to remove pebbles and large organic debris, while the remainder was unsieved for a soil biological ana­lysis. Samples were kept in sterile plastics bags and stored in a refrigerator at 4°C for further biological ana­lyses. The coordinates of each sample collection point were obtained using a global positioning system (GPS).

Twenty-one samples were collected from the rhizospheres of agricultural plants, mainly soybean (*Glycine max merril*), paddy rice (*Oryza sativa*), long bean (*Vigna sinensis*), corn (*Zea mays*), squash (*Luffa acutangula*), cacao (*Theobrema cacao*), chili (*Capsicum anuum*), kangkong (*Ipomoea aquatic*), eggplant (*Solanum tuberosum*), cassava (*Manihot utilisina*), and peanut (*Arachis hypogea*).

### Isolation and identification of PSMs

PSMs were isolated from soil samples collected from each rhizosphere using serial dilutions and the agar pour plate method. Ten grams of soil samples from all rhizosphere sampling points was dispersed in 90‍ ‍mL of sterile distilled water and thoroughly shaken. A 1-mL aliquot was transferred using a sterile pipette to 9‍ ‍mL of sterile distilled water in a test tube and stirred for 10‍ ‍s to form a 10^–2^ dilution. Serial dilutions to 10^–7^ were then prepared using the same method. A 0.1-mL of aliquot from each serial dilution was transferred to a sterile plate and covered with Pikovskayas agar medium (50°C) containing insoluble tricalcium phosphate, followed by an incubation at 27–30°C for 7 days. The composition of the medium was 5‍ ‍g Ca_3_(PO_4_), 0.5‍ ‍g (NH_4_)_2_SO_4_, 0.2‍ ‍g NaCl, 0.1‍ ‍g MgSO_4_.7H_2_O, 0.2‍ ‍g KCl, 10‍ ‍g glucose, 0.5‍ ‍g yeast extract, 20‍ ‍g agar, small amounts of MnSO_4_ and FeSO_4_, and 1,000‍ ‍mL distilled water (Subba Rao, 1982). Colonies with clear halos (a sign of solubilization) were considered to be phosphate-solubilizing colonies (Subba Rao, 1977; Vyas *et al.*, 2007). The number of viable cells was calculated using the following formula: number of cells mL^–1^ (CFU g^–1^)=(number of colonies)×(dilution factor) (Cappuccino and Sherman, 1992). Colonies surrounded by a halo zone were then transferred to Pikovskayas agar medium to maintain the purity of the culture for morphological, physiological, and biochemical ana­lyses as well as microbial identification. PSM isolates was identified based on colony and cell morphologies as well as microscopic observations using Bergey’s Manual of Systematic Bacteriology (Krieg and Holt, 1984).

### Phosphate-solubilizing index (PSI)

Halozone and colony diameters were successively measured during the incubation period to assess the PSI of PSMs. The PSI is the ratio of the total diameter, *i.e.* the clearance zone, including bacterial growth, and the diameter of the colony.

### Soil ana­lysis

Composite soil samples from all locations were air-dried, crushed, and passed through a 2-mm sieve and then analyzed for soil properties, such as pH related to soil-water 1:5 (w/v) (H_2_O 1:5), available phosphorus (mg kg^–1^) (Olsen, spectrophotometer), C-organic (g kg^–1^) (Walkley and Black, 1934, spectrophotometer), and total nitrogen (g kg^–1^) (Kjeldahl Method, spectrophotometer), while the soil moisture content (%) (gravimetric method) was measured using a fresh soil sample. Data on the population of PSMs and PSI were analyzed using the *t*-test. The relationships between PSMs and soil characteristics were examined using Pearson’s correlation (SPSS).

## Results

### Population of PSMs

A significant difference was observed in the population of PSMs between plant rhizospheres in agricultural soil (Table 1). The total population of PSB in soil samples from the Prafi and Masni Districts ranged between 25×10^3^ and 55×10^4^ CFU g^–1^ soil, while that of PSF ranged between 2.0×10^3^ and 5.0×10^3^ CFU g^–1^ soil. The highest number of PSB was found in the rhizosphere of cacao (*Theobrema cacao*), followed by chili (*Capsicum anuum*) and corn (*Zea mays*) in Masni District, while the lowest was detected in the rhizosphere of squash (*Luffa acutangula*) in Prafi District. The total population of PSF was low in most rhizosphere samples. In comparisons between Prafi and Masni Districts, the total populations of PSB and PSF were low in the former. Prafi District is known as the center of agricultural production in Manokwari, most of the lands are cultivated with agricultural crops, and, thus, the use of chemical fertilizers and pesticides is more intense in this area.

### Identification and morphological and physiological characteristics of isolates

**Fig. 1.**
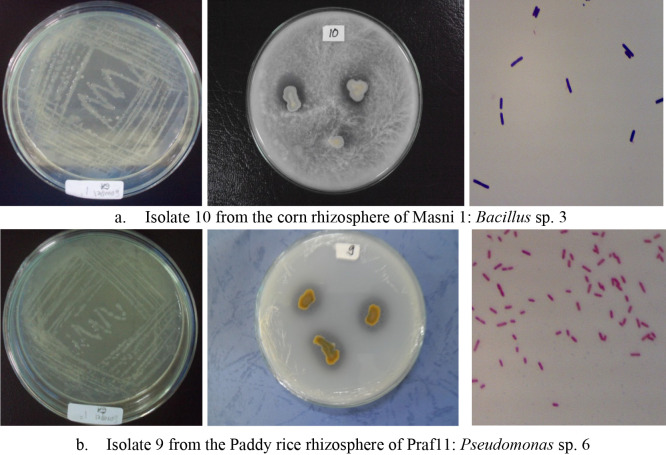
Isolates of phosphate-solubilizing microorganisms: (a) Isolate 10: *Bacillus* sp. 3 from Masni Districts in Manokwari. (b) Isolate 9: *Pseudomonas* sp. 6 from Prafi Districts in Manokwari.

Colony shapes were mostly circular/round (31 isolates) and whitish in color (32 isolates), while cell shapes were mainly rods (coccobacillus) (32 isolates) that were Gram negative (24 isolates) or Gram positive (12 isolates), and the majority of isolates were motile (Table 2). Six genera were identified among PSMs (5 PSBs and 1 PSF): *Chromobacterium* sp. (3 strains), *Pseudomonas* sp. (17 strains), *Bacillus* sp. (8 strains), *Micrococcus* sp. (5 strains), *Caulobacter* sp. (3 strains), and *Aspergillus* sp. (1 strain). The representative PSM types found in the agricultural soils of Prafi and Masni Districts are shown in Fig. 1.

### PSI

Approximately 58 PSM isolates were found in all sampling locations; however, only 37 PSM isolates, which considered as best PSM isolates, were identified and tested for their PSI (Table 3). The PSI of the isolates ranged between 1.1 and 3.6‍ ‍mm, with the most efficient and highest PSI being obtained for *Bacillus* sp. (strain 8) and the lowest for *Pseudomonas* sp. (strain 15).

However, among the isolates of *Pseudomonas* sp., six showed higher PSI than the others.

In the present study, an increase in the halo zone was not associated with a larger colony diameter.

### Soil characteristics

An ana­lysis of soil properties at each location examined (Table 4) revealed that soil pH varied between 5.20 and 6.40 (acidic to slightly acidic) in Prafi District and between 5.30 and 6.90 (acidic to neutral) in Masni District. The total nitrogen content ranged between 1.07‍ ‍g kg^–1^ (low) and 2.95‍ ‍g kg^–1^ (medium) in Prafi District, while that at all sampling locations in Masni District was low (0.14–0.16‍ ‍g kg^–1^). The phosphorus content in all areas was low to medium (6.00–9.90‍ ‍mg kg^–1^), while the carbon organic content was very low to low (0.10 to 0.28‍ ‍g kg^–1^) in Prafi District and low (1.20 to 1.91‍ ‍g kg^–1^) in Masni District. Prafi and Masni Districts are the main centers of agricultural crop production for vegetables and food crops in Manokwari region and Papua Barat Province; therefore, most of the agricultural land is highly dependent on chemical fertilizers and pesticides, the high and continuous use of which without the application of organic matter has affected the physical, chemical, and biological properties of soil.

### Relationship between PSMs and soil properties

Correlations were observed between the number of PSMs and soil P availability and moisture content, indicating an increase in soil P availability with a greater abundance of PSMs (Table 5). However, no correlations were noted with other soil characteristics, such as soil pH, N-total, and C-organic.

## Discussion

PSM populations were abundant and varied in most of the agriculture soil samples collected from different plant and soil rhizospheres in Prafi and Masni Districts. This result is consistent with previous findings reported by Kucey (1983) and Baliah *et al.* (2016), showing that the population of PSMs varied within the soil rhizosphere and with soil characteristics. However, in the present study, PSM populations were smaller in Prafi and Masni agricultural soil samples than in other types of soil containing high organic matter and soil nutrients, in which the population ranged between 10^4^ and 10^6^ CFU g^–1^. Although PSMs are found in all soils, their number depended on the soil climate as well as cropping history (Gupta *et al.*, 1986). The number of PSB was found to higher than that of PSF in Prafi and Masni Districts. Bacteria are more effective at phosphorus solubilization than fungi (Alam *et al.*, 2002). According to Chen *et al.* (2006), among the whole microbial population in soil, PSB are responsible for between 1 and 50% of the P solubilization potential and PSF for only 0.1 to 0.5%. Khan *et al.* (2009) reported that 1‍ ‍g of fertile soil contained between 10^1^ and 10^10^ bacteria and their live weight may exceed 2,000‍ ‍kg ha^–1^. Yahya and Azawi (1998) also showed that the abundance of PSB was generally large in agricultural and rangeland soils. PSB have been detected in the majority of soils (Chhonka and Taraedar, 1984), and their population is generally low in arid and semi-arid regions, which is attributed to the low level of organic matter and high temperature regime (Gupta *et al.*, 1986). The PSB population was found to be higher in soils in mild and moist climates than those in dry climates (Subba Rao, 1982). Different plant species or genotypes are another factor that affected the number and activity of soil microorganisms in the present study. This is consistent with the findings reported by Katiyar and Goel (2003) showing that the abundance of PSB in soil depended on the plant species, the microbial composition in soil, and soil conditions, in addition to the location of sampling (Kundu *et al.*, 2009). Belimov *et al.* (2015) and Schreiter *et al.* (2014) demonstrated that the diversity of microorganisms in different plant species may be attributed to plant-microbe interactions being highly dependent on soil conditions and the plant genotype. Furthermore, Baliah *et al.* (2016) found that the population level of PSB varied in the rhizosphere soils of okra, chili, tomato, cotton, and eggplant. Ponmurugan and Gopi (2006) reported that the PSB population was the largest in the rhizosphere soil of groundnut and the lowest in the rhizosphere soils of ragi, sorghum, and maize, and suggested that this was due to high phosphatase activity in the rhizosphere. Another factor contributing to variations in the PSB population may be the development of microorganisms in soil that are strongly influenced by the metabolic activity of plant roots through root exudates. Kato *et al.* (1997) showed that metabolic activity and metabolite compounds released by plants through the roots had a marked impact on the soil microorganisms present in plant root areas; therefore, soil microorganism activity will increase in the rhizosphere. In addition, variations in the PSB population of different crops were attributed to soil factors, such as nutrients, pH, moisture content, organic matter, and some soil enzyme activities for each crop (Ponmurugan and Gopi, 2006).

However, another study reported no relationship between the number of PSB and the type of vegetation (Kucey, 1983) or sampled sites and soil management programs (Fernández *et al.*, 2015). Another factor affecting the population of PSMs in soil is soil properties, *i.e.* physical, chemical, and biological soil properties. In the present study, no correlations were observed between the population of PSMs and some of the soil characteristics analyzed (pH, total N, and C-organic). In contrast, a correlation was found between the number of PSB and total nitrogen and organic matter (Rfaki *et al.*, 2017); differences in the organic carbon content in soil affected the distribution of PSB (Yadav and Singh, 1991); and a high content of soil organic matter increases productive soil microbes (John *et al.*, 2001) including PSB. In addition, the intensive and excessive use of chemical fertilizers and pesticides in agricultural soils affects soil microorganisms and PSMs. Adul Rahman *et al.* (2021) reported that any external pressure, including chemicals, affects the chemistry and physics of soil and, thus, its living organisms. Among PSM types, the six PSM isolates identified in the present study have been widely reported for PSB in previous studies with isolation sources ranging from rhizosphere soils (Gupta *et al.*, 2012; Singh and Prakash, 2012) to other common soils (Baliah *et al.*, 2016). The genus *Aspergillus* was the most frequent PSF isolate reported (Gupta *et al.*, 2007; Khan *et al.*, 2010; Simfukwe and Tindwa, 2018).

## Conclusions

The number and type of PSMs in the agricultural soils of Prafi and Masni Districts revealed that these areas are suitable habitats and these microorganisms may increase the P content in soil as well as its supply to crops. Correlations were observed between the number of PSMs and the level of soil P availability and moisture content, indicating an increase in soil P availability with a greater abundance of PSMs in soil.

## Citation

Djuuna, I. A. F., Prabawardani, S., and Massora, M. (2022) Population Distribution of Phosphate-solubilizing Microorganisms in Agricultural Soil. *Microbes Environ ***37**: ME21041.

https://doi.org/10.1264/jsme2.ME21041

## Figures and Tables

**Table 1.** Population of phosphate-solubilizing microorganisms (bacteria and fungi) in rhizospheres of agricultural crops in Prafi and Masni Districts, Manokwari

Sampling Site	Coordinate			Crops			Mean Population of PSM (CFU g^–1^ soil)×10^3^	
	North (X)	East (Y)		Common Name	Botanical Name		Bacteria	Fungi
PRAFI								
Prafi 1	360157	9911265		Chili	*Capsicum anuum*		540	3.0
Prafi 1	359535	9911392		Kangkung	*Ipomoea aquatica*		330	2.0
Prafi 1	358187	9911891		Paddy Rice	*Oryza sativa*		500	2.0
Prafi 1	363571	9906585		Squash	*Luffa acutangula*		25	5.0
Prafi 2	363700	9905520		Soybean	*Glycine max*		34	3.0
Prafi 2	364130	9904922		Eggplant	*Solanum tuberosum*		47	2.0
Prafi 2	364469	9904450		Cassava	*Manihot utilisina*		32	2.0
Prafi 3	368901	9902105		Corn	*Zea mays*		420	5.0
Prafi 3	368579	9901765		Cacao	*Theobrema cacao*		51	3.0
Prafi 3	368537	9901041		Cassava	*Manihot utilisina*		43	2.0
Prafi 4	370247	9901060		Peanut	*Arachis hypogea*		59	2.0
Prafi 4	370715	9901748		Long beans	*Vigna sinensis*		370	5.0
Prafi 4	371477	9901584		Cacao	*Theobrema cacao*		550	3.0
MASNI								
Masni 1	375397	9898650		Cacao	*Theobrema cacao*		16	3.0
Masni 1	376286	9899340		Corn	*Zea mays*		520	3.0
Masni 2	377516	9897641		Cacao	*Theobrema cacao*		100	2.0
Masni 2	376932	9897807		Corn	*Zea mays*		19	2.0
Masni 2	376832	9897370		Kangkong	*Ipomoea aquatica*		390	5.0
Masni 2	376902	9897444		Paddy rice	*Oryza sativa*		480	2.0
Masni 3	388221	9890357		Paddy rice	*Oryza sativa*		350	5.0

*t*-test: PSB: t=5.523 df=19 Sign. (2 tailed)=0.001  PSF: t=11.168 df=19 Sign. (2 tailed)=0.001

**Table 2.** Phosphate-solubilizing index of 37 PSM isolates from rhizosphere samples collected in Prafi and Masni Districts

Isolate	Halozone Diameter (mm)	Colony Diameter (mm)	Phosphate-solubilizing Index (mm)	Species/Strain
1	23	12	1.9	*Chromobacterium* sp. (1)
2	19	10	1.9	*Pseudomonas* sp. (1)
3	17	10	1.7	*Pseudomonas* sp. (2)
4	17	8	2.1	*Pseudomonas* sp. (3)
5	14	8	1.7	*Bacillus* sp. (1)
6	15	8	1.9	*Pseudomonas* sp. (4)
7	16	8	2.0	*Bacillus* sp 2
8	12	7	1.7	*Pseudomonas* sp. (5)
9	12	11	1.1	*Pseudomonas* sp. (6)
10	10	7	1.4	*Bacillus* sp. (3)
11	13	8	1.6	*Pseudomonas* sp. (7)
12	13	6	2.2	*Pseudomonas* sp. (8)
13	10	6	1.7	*Bacillus* sp. (4)
14	11	7	1.6	*Bacillus* sp. (5)
15	12	6	2.0	*Bacillus* sp. (6)
16	15	12	1.2	*Chromobacterium* sp. (2)
17	15	11	1.4	*Pseudomonas* sp. (9)
18	13	7	1.8	*Pseudomonas* sp. (10)
19	14	6	2.3	*Micrococcus* sp. (1)
20	11	8	1.4	*Chromobacterium* sp. (3)
21	21	9	2.3	*Pseudomonas* sp. (11)
22	22	9	2.4	*Pseudomonas* sp. (12)
23	3	2	1.5	*Pseudomonas* sp. (13)
24	8	5	1.6	*Bacillus* sp. (7)
25	11	7	1.6	*Micrococcus* sp. (2)
26	18	5	3.6	*Bacillus* sp. (8)
27	15	10	1.5	*Pseudomonas* sp. (14)
28	13	8	1.6	*Caulobacter* sp. (1)
29	11	6	1.8	*Caulobacter* sp. (2)
30	14	11	1.3	*Micrococcus* sp. (3)
31	9	7	1.3	*Micrococcus* sp. (4)
32	13	11	1.1	*Pseudomonas* sp. (15)
33	17	7	2.4	*Pseudomonas* sp. (16)
34	11	7	1.8	*Micrococcus* sp. (5)
35	14	11	1.3	*Caulobacter* sp. (3)
36	13	6	2.2	*Pseudomonas* sp. (17)
37	10	7	1.4	*Aspergillus* sp. (1)

*t*-test for PSI: t=22.654 df=36 Sign. (2 tailed)=0.001 Numbers in parentheses show the strains of PSB

**Table 3.** Morphology, physiology, and biochemical activities of isolates of phosphate-solubilizing microorganisms

Isolate	Morphology						Physiology										Biochemical Activities																		Strain
	Colony			Cell			Temperature (°C)						pH				Motility	Catalase	Oxidase	MR	VP	Indole	Citrate	Urease	Nitrate Reduction	H_2_S Production	Starch Hydrolysis	Gelatin Hydrolysis	Utilization of Glucose	Utilization of β-Alanine	Utilization of L-Arginine	Utilization of D-Xylose	Utilization of D-Ribose	Utilization of Sucrose	
	Color	Shape		Shape	Gram Stain		15	25	37	45	50		4	5	6	7																			
1	Reddish brown	Circular		Rod	Negative		+	+	+	+	–		–	–	+	+	+	+	+	+	–	–	–	–	+	–	–	+	+	–	+	–	–	+	*Chromobacterium* sp. 1
2	Whitish	Rhizoid		Rod	Negative		+	+	+	+	–		–	+	+	+	+	+	+	–	–	–	+	–	+	–	–	+	+	+	+	–	+	–	*Pseudomonas* sp. 1
3	Yellow	Circular		Rod	Negative		+	+	+	+	–		+	+	+	+	+	+	+	–	–	–	+	–	+	–	–	+	+	+	+	–	–	–	*Pseudomonas* sp. 2
4	Whitish	Circular		Rod	Negative		+	+	+	+	–		–	+	+	+	+	+	+	–	–	–	+	–	+	–	–	+	+	+	+	+	+	–	*Pseudomonas* sp. 3
5	Whitish	Circular		Rod	Positive		+	+	+	+	+		+	+	+	+	+	+	+	–	+	–	+	–	+	–	+	+	+	–	–	–	+	+	*Bacillus* sp. 1
6	Whitish	Circular		Rod	Negative		+	+	+	+	–		–	+	+	+	+	+	+	–	–	–	+	–	+	–	–	+	+	+	+	–	+	–	*Pseudomonas* sp. 4
7	Whitish	Circular		Rod	Positive		+	+	+	+	+		+	+	+	+	+	+	+	–	+	–	+	–	+	–	+	+	+	–	–	–	+	+	*Bacillus* sp. 2
8	Whitish	Circular		Rod	Negative		+	+	+	+	–		–	+	+	+	+	+	+	–	–	–	+	–	+	–	–	+	+	+	+	–	+	–	*Pseudomonas* sp. 5
9	Whitish	Circular		Rod	Negative		+	+	+	+	–		–	+	+	+	+	+	+	–	–	–	+	–	+	–	–	+	+	+	+	–	+	–	*Pseudomonas* sp. 6
10	Whitish	Circular		Rod	Positive		+	+	+	+	+		+	+	+	+	+	+	+	–	+	–	+	–	+	–	+	+	+	–	–	–	+	+	*Bacillus* sp. 3
11	Whitish	Irregular		Rod	Negative		+	+	+	+	–		–	+	+	+	+	+	+	–	–	–	+	–	+	–	–	+	+	–	+	–	+	–	*Pseudomonas* sp. 7
12	Whitish	Circular		Rod	Negative		+	+	+	+	–		–	+	+	+	+	+	+	–	–	–	+	–	+	–	–	+	+	+	+	–	+	–	*Pseudomonas* sp. 8
13	Whitish	Circular		Rod	Positive		+	+	+	+	+		–	+	+	+	+	+	+	–	+	–	+	–	+	–	+	+	+	–	–	–	+	+	*Bacillus* sp. 4
14	Whitish	Circular		Rod	Positive		+	+	+	+	+		–	+	+	+	+	+	+	–	+	–	+	–	+	–	+	+	+	–	–	–	+	+	*Bacillus* sp. 5
15	Whitish	Circular		Coccus	Positive		+	+	+	+	+		–	+	+	+	+	+	+	–	+	–	+	–	+	–	+	+	+	–	–	–	+	+	*Bacillus* sp. 6
16	Whitish	Circular		Rod	Negative		+	+	+	+	–		–	+	+	+	+	+	+	+	–	–	–	–	+	–	–	+	+	–	+	–	–	+	*Chromobacterium* sp. 2
17	Whitish	Circular		Rod	Negative		+	+	+	+	–		–	+	+	+	+	+	+	–	–	–	+	–	+	–	–	+	+	+	+	–	+	–	*Pseudomonas* sp.
18	Whitish	Circular		Rod	Negative		+	+	+	+	–		–	+	+	+	+	+	+	–	–	–	+	–	+	–	–	+	+	+	+	–	+	–	*Pseudomonas* sp. 10
19	Whitish	Circular		Rod	Positive		–	+	+	–	–		–	+	+	+	–	+	+	+	–	–	–	–	–	–	+	+	–	+	–	–	–	+	*Micrococcus* sp. 1
20	Whitish	Circular		Rod	Negative		+	+	+	+	–		–	+	+	+	+	+	+	+	–	–	–	–	+	–	–	+	+	–	+	–	–	+	*Chromobacterium* sp. 3
21	Whitish	Irregular		Coccus	Negative		+	+	+	+	–		–	+	+	+	+	+	+	–	–	–	+	–	+	–	–	+	+	+	+	+	+	–	*Pseudomonas* sp. 11
22	Whitish	Circular		Rod	Negative		+	+	+	+	–		–	+	+	+	+	+	+	–	–	–	+	–	+	–	–	+	+	+	+	–	+	–	*Pseudomonas* sp. 12
23	Whitish	Circular		Rod	Negative		+	+	+	+	–		–	+	+	+	+	+	+	–	–	–	+	–	+	–	–	+	+	+	+	–	+	–	*Pseudomonas* sp. 13
24	Whitish	Circular		Rod	Positive		+	+	+	+	+		–	+	+	+	+	+	+	–	+	–	+	–	+	–	+	+	+	–	–	–	+	+	*Bacillus* sp. 7
25	Whitish	Circular		Rod	Positive		–	+	+	–	–		–	+	+	+	–	+	+	+	–	–	–	–	–	–	+	+	–	+	–	–	–	+	*Micrococcus* sp. 2
26	Whitish	Circular		Rod	Positive		+	+	+	+	+		–	+	+	+	+	+	+	–	+	–	+	–	+	–	+	+	+	–	–	–	+	+	*Bacillus* sp. 8
27	Whitish	Circular		Rod	Negative		+	+	+	+	–		–	+	+	+	+	+	+	–	–	–	+	–	+	–	–	+	+	+	+	–	+	–	*Pseudomonas* sp. 14
28	Whitish	Circular		Rod	Negative		–	+	+	–	–		–	–	+	+	+	+	+	+	–	–	+	+	+	–	–	–	+	+	+	–	+	+	*Caulobacter* sp. 1
29	Whitish	Circular		Rod	Negative		–	+	+	–	–		–	–	+	+	+	+	+	+	–	–	–	+	–	–	–	–	+	+	+	–	+	+	*Caulobacter* sp. 2
30	Whitish	Circular		Coccus	Positive		–	+	+	–	–		–	+	+	+	–	+	+	+	–	–	–	–	–	–	+	+	–	+	–	–	–	+	*Micrococcus* sp. 3
31	Whitish	Circular		Coccus	Positive		–	+	+	–	–		–	+	+	+	–	+	+	+	–	–	–	–	–	–	+	+	–	+	–	–	–	+	*Micrococcus* sp. 4
32	Whitish	Irregular		Rod	Negative		+	+	+	+	–		–	+	+	+	+	+	+	–	–	–	+	–	+	–	–	+	+	+	+	–	+	–	*Pseudomonas* sp. 15
33	Whitish	Rhizoid		Rod	Negative		+	+	+	+	–		–	+	+	+	+	+	+	–	–	–	+	–	+	–	–	+	+	+	+	–	+	–	*Pseudomonas* sp. 16
34	Whitish	Irregular		Rod	Positive		–	+	+	–	–		–	+	+	+	–	+	+	+	–	–	–	–	–	–	+	+	–	+	–	–	–	+	*Micrococcus* sp. 5
35	Transparent	Circular		Rod	Negative		–	+	+	–	–		–	–	+	+	+	+	+	+	–	–	–	+	+	–	–	–	+	+	+	–	+	+	*Caulobacter* sp. 3
36	Whitish	Circular		Rod	Negative		+	+	+	+	–		–	+	+	+	+	+	+	–	–	–	+	–	+	–	–	+	+	+	+	–	+	–	*Pseudomonas* sp. 17
37	Brownish	Circular					–	+	+	–	–		–	–	+	+																			*Aspergillus* sp. 1

MR=Methyl Red; VP=Voges-Proskauer

**Table 4.** Soil characteristics at each sampling site of agricultural soil in Prafi and Masni Districts, Manokwari

Sampling site	Soil Characteristics				
	pH H_2_O (1:5)	Moisture Content (%)	N-Total (g kg^–1^)	Available P (mg kg^–1^)	C-Org (g kg^–1^)
PRAFI					
Prafi 1 (1)	6.3 SA	31.0	12 L	7.6 M	1.4 VL
Prafi 1 (2)	6.4 SA	24.0	12 L	6.6 L	1.3 VL
Prafi 2 (1)	6.3 SA	26.9	21.5 L	7.5 M	2.0 VL
Prafi 3 (1)	5.2 A	20.8	29.5 M	9.9 M	2.8 VL
Prafi 3 (2)	6.4 SA	28.3	10.7 L	6.3 L	1.0 VL
Prafi 4 (1)	5.8 SA	15.9	13.5 L	7.1 M	1.4 VL
Prafi 4 (2)	6.1 SA	20.7	24.7 M	8.4 M	2.5 VL
MASNI					
Masni 5 (1)	5.3 A	27.5	15 L	6.0 L	17.5 L
Masni 5 (2)	5.9 SA	17.8	16 L	7.1 M	19.1 L
Masni 6 (1)	6.1 SA	18.4	16 L	7.9 M	16.0 L
Masni 6 (2)	6.9 N	35.3	15 L	7.6 M	13.5 L
Masni 7 (1)	6.0 SA	32.6	14 L	6.8 L	12.0 L

VL=Very Low; L=Low; M=Medium; A=Acid; SA=Slightly Acidic; N=Neutral

**Table 5.** Correlation matrices between populations of PSMs and soil characteristics

Variables	Water Content	pH	C organic	N total	P	PSB	PSF
Water Content	1						
pH	0.546**	1					
C	–0.371	0..491	1				
N	–0.301	–0.523*	0.971**	1			
P	–0.067	0.291	0.134	0.145	1		
PSB	0.694*	0.220	0.139	0.184	0.641**	1	
PSF	0.084	–0.100	0.676**	0.190	0.070	0.891**	1
